# Mechanism for Adhesion G Protein-Coupled Receptor GPR56-Mediated RhoA Activation Induced By Collagen III Stimulation

**DOI:** 10.1371/journal.pone.0100043

**Published:** 2014-06-20

**Authors:** Rong Luo, Sung-Jin Jeong, Annie Yang, Miaoyun Wen, David E. Saslowsky, Wayne I. Lencer, Demet Araç, Xianhua Piao

**Affiliations:** 1 Division of Newborn Medicine, Boston Children’s Hospital and Harvard Medical School, Boston, Massachusetts, United States of America; 2 Division of Gastroenterology, Boston Children's Hospital, Boston, Massachusetts, United States of America; 3 Department of Biochemistry and Molecular Biology, University of Chicago, Chicago, Illinois, United States of America; Wayne State University, United States of America

## Abstract

GPR56 is a member of the adhesion G protein-coupled receptor (GPCR) family. Despite the importance of GPR56 in brain development, where mutations cause a devastating human brain malformation called bilateral frontoparietal polymicrogyria (BFPP), the signaling mechanism(s) remain largely unknown. Like many other adhesion GPCRs, GPR56 is cleaved via a GPCR autoproteolysis-inducing (GAIN) domain into N- and C-terminal fragments (GPR56^N^ and GPR56^C^); however, the biological significance of this cleavage is elusive. Taking advantage of the recent identification of a GPR56 ligand and the presence of BFPP-associated mutations, we investigated the molecular mechanism of GPR56 signaling. We demonstrate that ligand binding releases GPR56^N^ from the membrane-bound GPR56^C^ and triggers the association of GPR56^C^ with lipid rafts and RhoA activation. Furthermore, one of the BFPP-associated mutations, L640R, does not affect collagen III-induced lipid raft association of GPR56. Instead, it specifically abolishes collagen III-mediated RhoA activation. Together, these findings reveal a novel signaling mechanism that may apply to other members of the adhesion GPCR family.

## Introduction

Adhesion G protein-coupled receptors (GPCRs) are a family of noncanonical seven transmembrane spanning (7TM) receptors. There are a total of 33 members in the family in both humans and mice, present in almost every organ system with physiological functions in development, reproduction, immunity, neuronal and epithelial function, as well as tumorigenesis [1]. Structurally, they are differentiated from other subgroups of GPCRs by the presence of an exceptionally long extracellular N-terminal region and juxtamembrane GPCR autoproteolysis-inducing (GAIN) domain [2]–[4]. Most members of the adhesion GPCRs undergo GAIN domain-mediated autoproteolytic process at the GPCR proteolysis site (GPS) motif to produce an N-terminal fragment and a C-terminal fragment [4], [5]. The biological significance of this autocleavage and its implication in receptor signaling remain largely unknown.

GPR56 is one important member of the adhesion GPCR family, as mutations in GPR56 cause a devastating human brain malformation called bilateral frontoparietal polymicrogyria (BFPP) [6], [7]. Additionally, GPR56 has also been reported to play a critical role in cancer progression by regulating angiogenesis [8], [9]. We recently discovered that collagen III is a ligand of GPR56 in the developing brain and that the binding of GPR56 to collagen III activates RhoA by coupling to Gα_12/13_ [10]. In the context of cancer biology, GPR56 was shown to bind tissue transglutaminase (TG2). Although it is unclear whether the binding of TG2 to GPR56 triggers downstream signaling, deleting the binding site of TG2 in GPR56 activates PKCα and elevates VEGF production in a melanoma cell line MC-1 [8], [9]. Nevertheless, the molecular mechanism(s) underlying GPR56 signaling, including the importance of GPR56^N^-GPR56^C^ interactions, remain poorly understood.

To gain insight into GPR56 signaling, we explored the molecular mechanism of the activation of GPR56 signaling by collagen III using wild type GPR56 and its BFPP associated mutants. Our results demonstrate that collagen III binding causes the release of GPR56^N^ from cell surfaces and induces GPR56^C^ redistribution to detergent resistant membrane fragments (DRMs), the biochemical correlate of lipid rafts. Furthermore, L640 is an evolutionarily conserved amino acid in GPR56 across multiple species, and a BFPP-associated mutation at this amino acid residue, L640R, specifically abolishes collagen III-induced RhoA activation.

## Materials and Methods

### Reagents and Antibodies

Sulfo-NHS-Biotin reagent was purchased from Pierce; Mem-PER Eukaryotic Membrane Protein Extraction Reagent Kit was from Thermo Scientific; Streptavidin–agarose beads from Sigma; RIPA buffer from Boston Bioproducts; Protease inhibitor cocktail (EDTA-free) from Roche Diagnostics; Collagen III protein was from AbCam; Mouse GPR56 cDNA cloned into pCDNA3.1(+) vector as described previously [11]; GPR56 mutations were created by site-directed mutagenesis using the QuikChange II XL Site-Directed Mutagenesis kit (Stratagene), as previously described [12]; Cholera toxin B subunit (CTB)–Alexa Fluor 488 were purchased from Invitrogen. Rabbit polyclonal anti-CTB antibody was generated in the Lencer lab [13]. HRP-labeled secondary antibodies were purchased from Sigma-Aldrich. Alexa Fluor 488 goat anti-mouse IgG (H+L) was purchase from Invitrogen. Mouse anti-GPR56^N^ (CG4) was purchased from Biolegend. The mouse anti-GPR56^N^ (H11) antibody was generated at the Dana Farber/Harvard Cancer Center Monoclonal Antibody Core and the rabbit anti-GPR56^C^ (199) antibody was generated at Yenzym Antibodies, as previously described [14], [15]. The GST-RBD beads and mouse monoclonal anti-RhoA antibody were purchased from Cytoskeleton.

### Cells

SH-SY5Y cells uptake limited copy number of transgene during *in vitro* transient transfection, which is more relevant to the *in vivo* protein expression pattern. Therefore, we used SH-SY5Y cells for all imaging and flow cytometry study. However, this cell line is not suitable for analyses that require high transfection efficiency, such as DRM fractionation, Co-IP, GTP-Rho Pull-Down Assay, and Western blot analysis of the cell conditioned media. Thus, we used HEK 293T cells for those assays.

### Biotinylation of Cell Surface Proteins and Western Blot

Biotinylation of cell surface proteins was performed as previously described [12]. HEK293T cells were transfected with VSVG/His-tagged wild-type or mutant GPR56 constructs. Biotinylated proteins were enriched with streptavidin agarose beads, followed by western blot with standard western blot protocol. Western blot band density in each experiment was measured by Image QuantTL (Amersham Bioscience, Arlington Heights, IL). Two-tailed Student’s t-tests were performed for P values.

### Membrane Protein Extraction and Co-Immunoprecipitation (co-IP)

The membrane proteins were enriched by Mem-PER Eukaryotic Membrane Protein Extraction Reagent Kit (Thermo Scientific), following manufactory protocol. Briefly, HEK 293T cells transfected with wild type or L640R mutant *Gpr56* cDNA were stimulated with collagen III or the vehicle (acetic acid) for 5 minutes, as described in previously [10]. About 5×10^6^ cells per sample were used for membrane protein extraction. The isolated membrane fraction samples were precleared for 1 h with protein G Sepharose (Invitrogen), followed by incubation with rabbit anti-GPR56^C^ (199) antibodies along with protein G-Sepharose. The immune complexes were washed and eluted with Laemmli Buffer for western blot. GPR56^N^ and GPR56^C^ proteins were detected by mouse anti-GPR56^N^ (H11) and rabbit anti-GPR56C (199), respectively.

### Immunofluorescent Confocal Imaging

For cell surface staining of GPR56^N^ and GPR56^C^, SH-SY5Y cells were plated on Poly-d-lysin coated cover glass and were transfected with wild type or L640R mutant *Gpr56* cDNA and cultured for 24 hr. The transfected cells were stimulated with collagen III or acetic acid as a control for 5 min. Cells were fixed in 2% PFA for 10 min, permeablized with 0.1% saponin for 5 min, incubated with rabbit- anti GPR56^C^ (199) or mouse-anti GPR56^N^ (H11) antibodies visualized by rabbit Alexa-546 and mouse Alexa-488, respectively. Images were captured by a confocal microscope.

### Lipid Raft Separation

HEK 293T cells transfected with wild type or L640R mutant *Gpr56* cDNA were incubated with 3 nM CTB for 50 minutes at 37°C, then stimulated with collagen III or the acetic acid for 5 minutes. All remaining steps were carried out on ice. Cells (∼10×10^6^ cells) were washed three times in ice-cold PBS and then lysed in 500 ul ice cold DEB buffer (10 mM Tris, 150 mM NaCl pH 7.5) containing 1% Triton X-100. Cells were homogenized using a loose-fitting Dounce homogenizer 10 times. The homogenate was then passed through a 25-gauge needle 10 times. The cell lysates were mixed with the same volume of 80% sucrose prepared in DEB buffer and placed at the bottom of an ultracentrifuge tube. A 5–30% linear sucrose gradient was formed above the homogenate and centrifuged at 37,000 rpm at 4°C for overnight in an SW41 rotor (Beckman Instruments). Fractions were carefully removed following centrifugation, combined into caveolin-enriched fractions. Following ultracentrifugation, gradient fractions (500 µL) were collected from top to bottom with fraction 11 corresponding to the bottom most fraction. Aliquots of each fraction were subjected to SDS-PAGE and western blot analysis.

### Flow Cytometry

SH-SY5Y cells were transfected with wild type or L640R mutant *Gpr56* cDNA and the transfected cells were cultured for 48 hours and followed by collagen III stimulation for 5 min. After washing with cold PBS and blocking in 5% goat serum, 0.5% BSA, 0.1% NaN_3_ in PBS and incubating for 30 min at 4°C, the cells were incubated with mouse anti-GPR56^N^ antibody (CG4, 1 in 50 dilution) for 30 min at 4°C, followed by a second incubation step with Alexa Fluor 488 goat anti-mouse IgG. The stained cells were washed with washing buffer (0.1% BSA, 0.1% NaN_3_ in PBS), and then fixed with 2% PFA. After 15 min fixation at room temperature, cells were washed with PBS and store in PBS buffer at 4°C until FACS. Flow cytometric analysis was performed using a FACSCalibur (BD Biosciences) and the FlowJo software package (Tree Star, Ashland, OR).

### Detection of the Released GPR56^N^ in Cell Conditioned Media

HEK 293T cells were transiently transfected with *Gpr56* cDNA. Forty eight hours later, the transfected cells were washed three times with PBS, followed by the treatment with acetic acid or collagen III for 5 minutes. The conditioned media were harvested, filtered, and concentrated as previously described [12]. Equal volume of the concentrated media were used for Western blot.

### GTP-Rho Pull-Down Assay

The GTP-Rho pull-down assay was done as previously described, using 293T cells transfected with either wild type or L640R mutant *Gpr56* cDNA [10]. The GST-RBD beads bound Rho proteins were boiled in Laemmli buffer and detected by Western blotting using mouse monoclonal anti-RhoA antibody.

## Results

### GPR56^N^ Remains Associated with GPR56^C^ on Plasma Membrane in Both Wild Type and Disease-causing Mutant Proteins

**Figure 1:**
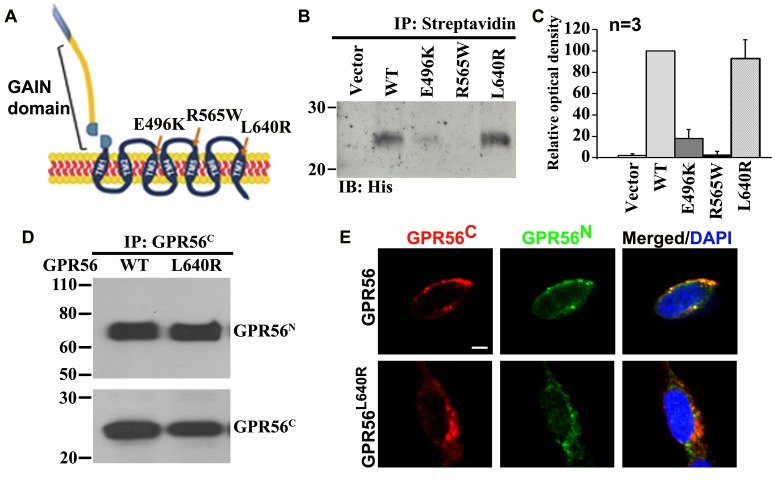
Cell surface expression of GPR56 and its mutant protein. (**A**). Schematic representation of GPR56 protein with three GPR56^C^ mutations indicated. (**B**). Detection of membrane expressed GPR56 by biotinylation experiments. Cells transfected with wild type *Gpr56* and L640R mutant cDNA have comparable cell surface expression of GPR56^C^, whereas cells transfected with either E496K or R565W show much reduced or absent cell surface expression of GPR56^C^. (**C**). Bar graphs depict optical density of the western blot of GPR56^C^ in (B). (**D**) Analysis of the N- and C- terminal fragment association on plasma membrane by coimmunoprecipitation (IP) experiments. The association of GPR56^N^ and GPR56^C^ was observed in both wild type and L640R mutant receptors. (**E**). Detection of colocalization of GPR56^N^ and GPR56^C^ on cell plasma membrane by immunostaining. Scale bar: 10 µm.

Among the reported disease-associated GPR56 mutant alleles, we have previously shown that mutations in the N-terminus of GPR56 abolish ligand binding and mutations in the GPS motif affect GAIN domain-mediated GPR56 protein cleavage [12], [16]. To further study the mechanism of the receptor signaling, we turned our attention to the three reported GPR56^C^ mutants, E496K, R565W, and L640R (Fig. 1A) [6], [7], [17]. We hypothesized that normally expressed BFPP-causing mutants could provide insight into GPR56 signaling. In order to identify mutations that do not affect the level of cell surface expression, we performed biotinylation experiments. HEK293T cells transfected with wild type or mutant GPR56 were labeled with sulfo-NHS-biotin, a membrane impermeable biotinylation agent. Thus, only cell surface-expressed proteins were labeled by biotin. The cells were lysed, and all biotinylated proteins were isolated by streptavidin affinity chromatography. Only the L640R mutant had a comparable level of GPR56^C^ expression as the wild type GPR56 and was selected for further studies (Fig. 1B and C).

We performed co-IP experiments with anti-GPR56^C^ antibody. As expected, GPR56^N^ remains associated with GPR56^C^ in both wild type and L640R mutant proteins (Fig. 1D). To further verify the association of GPR56^N^ and GPR56^C^ on the cell surface, we performed double immunostaining with antibodies against GPR56^N^ and GPR56^C^, respectively. As shown in Fig. 1E, cell surface expressed GPR56^N^ and GPR56^C^ were mostly co-localized in both wild type and L640R mutant proteins.

### Ligand Stimulation Triggers a Shift of GPR56^C^ from Non-DRM to DRM Fractions in both Wild Type and L640R Mutant GPR56

In recent decades, the original fluid mosaic model of the plasma membrane proposed by Singer and Nichols has been challenged [18]. Instead of a relatively continuous and homogenous fluid of amphipathic lipids interspersed with a mosaic of proteins, it has been found that the plasma membrane contains nanoscale domains of sphingolipids, cholesterol, and membrane proteins, which together form what is referred to as ‘lipid rafts’ that function as receptor signaling platforms [19], [20]. Lipid rafts are resistant to cold nonionic detergent treatment, causing them to float to the top fraction of isopycnic sucrose gradients; thus they are named detergent resistant membranes (DRMs) [19]–[21]. Proteins that associate with lipid rafts are defined as those that co-fractionate with DRM fractions. Therefore, cold-detergent extraction and membrane fractionation have been extensively used to identify proteins associated with lipid rafts.

**Figure 2:**
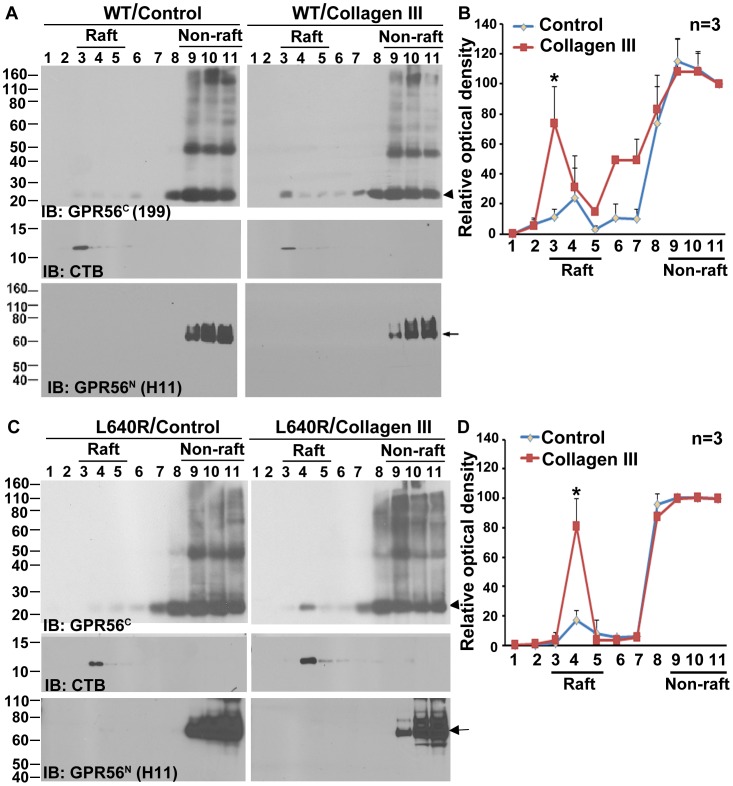
Collagen III induces a shift of GPR56^C^ from the non-raft to raft fractions. (**A**). Western blot analysis of the lipid raft fractionation of 293T cells transfected with wild type *Gpr56* using anti-GPR56^C^ (199), and GPR56^N^ (H11) to detect the C-terminal and N-terminal of GPR56, respectively. Cholera toxin B subunit (CTB), which binds to ganglioside GM1, served as a marker for lipid raft. Different lane numbers correspond to different fractions after sucrose gradient centrifugation. Higher protein bands in fraction 9–11 likely represent protein aggregate. Arrowhead indicating the corresponding GPR56^C^ and arrow showing the responding GPR56^N^. (**B**). The relative optical intensity of GPR56^C^ observed in A was measured using Image QuantTL program and presented as mean ± SE in a linear plot. n = 3, *P = 0.0124, Student *t* test. (**C**) Western blot analysis of the lipid raft fractionation of 293T cells transfected with L640R mutant cDNA. Higher protein bands in fraction 8–11 likely represent protein aggregate. Arrowhead indicating the corresponding GPR56^C^ and arrow showing the responding GPR56^N^. (**D**). The relative optical intensity of GPR56^C^ observed in C was measured using Image QuantTL program and presented as mean ± SE in a linear plot. n = 3, *P = 0.006, Student *t* test.

It was recently reported that a low level of GPR56^C^ is constitutively associated with membrane lipid rafts [22]. However, it is not known whether there is a dynamic presence of GPR56 in the lipid raft upon ligand stimulation. We set out to test the hypothesis that lipid raft association is required for GPR56 signaling. HEK 293T cells transfected with GPR56 cDNA were stimulated with either collagen III or acetic acid for 5 minutes. The cells were lysed in the presence of detergent (1% Triton X-100) on ice and subjected to DRM fractionation. GPR56^N^ is tethered non-covalently with GPR56^C^ on the plasma membrane, and therefore is restrictedly present in the non-raft fractions (Fig. 2A). Consistent with a previous report [22], we did detect a low basal level of GPR56^C^ in the lipid raft fractions (Fig. 2A). Interestingly, we observed a significant shift of GPR56^C^ from non-raft to lipid raft fractions upon collagen III stimulation (Fig. 2A and B), indicating that GPR56 probably needs lipid rafts as a platform for its signal transduction.

To examine whether the L640R mutation affects the GPR56^C^ shift to a lipid raft upon ligand stimulation, DRM analysis was performed using this mutant receptor. Our result showed that the mutant GPR56^C^ also translocated to lipid raft fractions after ligand stimulation (Fig. 2 C and D), similar to the behavior of wild type GPR56. This data indicated that this disease-associated C-terminal mutation does not disrupt collagen III-induced association of GPR56 with plasma membrane lipid nanodomains. Thus, collagen III binds both wild type and the L640R mutant, resulting in the C-terminal fragment associating with DRMs.

### Ligand Binding Releases GPR56^N^ from the Membrane-bound GPR56^C^

**Figure 3:**
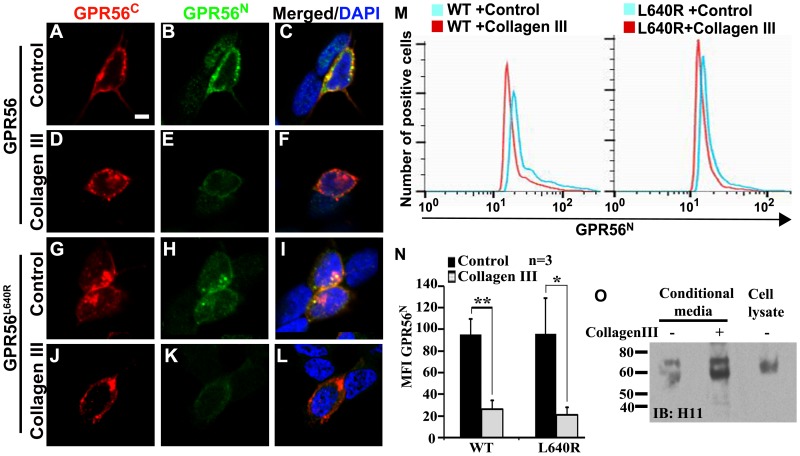
Collagen III treatment causes a reduced plasma membrane associated GPR56^N^. (**A–L**). Immunostaining of GPR56^N^ and GPR56^C^. Collagen III stimulation results in a decreased level of GPR56^N^. (**M**). Detection of surface expressed GPR56^N^ by flow cytometry. Surfaced expressed GPR56^N^ was probed with anti- GPR56^N^ (CG4) antibody, followed by flow cytometry analysis. Shown are representative histograms. (**N**) Bar graphs show the mean and SD of the geometric mean fluorescence intensity (MFI) of GPR56^N^ expression. n = 3, *P = 0.02, **P = 0.008. (**O**) Western blot detection of GPR56^N^ in the cell conditioned media with or without collagen III treatment. Collagen III treatment results in a higher GPR56^N^ in the cell conditioned media.

Overexpression of GPR56^C^ alone enhanced RhoA activation, suggesting that the binding of GPR56^N^ to GPR56^C^ probably inhibits GPR56 downstream signaling [23]. Therefore, we hypothesized that the binding of collagen III activates RhoA by removing GPR56^N^ from GPR56^C^. To test this hypothesis, we transfected *Gpr56* cDNA into SY5Y cells and stimulated the cells with collagen III or acetic acid as a control for 5 minutes. The cells were stained with antibodies against GPR56^C^ or GPR56^N^. There was no significant change in the level of plasma membrane wild type or L640R mutant GPR56^C^ upon collagen III binding as compared to controls (Fig. 3A, D, G, J), indicating that 5 min of stimulation does not induce GPR56^C^ endocytosis. Interestingly, membrane-bound GPR56^N^ was dramatically decreased upon collagen III treatment for both wild type and the L640R mutant (Fig. 3B, E, H, K). To quantify the reduction of cell surface GPR56^N^ after collagen III treatment, we performed flow cytometry analysis. Indeed, compared to the control and consistent with panels A–L, collagen III stimulation caused a significant reduction of surface GPR56^N^ in both wild type and L640 mutant (Fig. 3M and N). To exclude the possibility that GPR56^N^ was internalized upon binding to collagen III, we subjected the cell conditioned media to western blot analysis. As shown in Fig. 3O, higher level of GPR56^N^ was detected in collagen III treated cell conditioned media, indicating that the binding of collagen III releases GPR56^N^ from cell plasma membrane. We have previously shown that membrane-bound GPR56^N^ has lower molecular weight due to lesser degree of glycosylation, whereas secreted GPR56^N^ is more heavily glycosylated and therefore has higher molecular weight [12]. Interestingly, collagen III treatment predominantly enriched the membrane-bound GPR56^N^ (Fig. 3O).

### Collagen III Stimulation does not Induce RhoA Activation in the L640R Mutant

**Figure 4:**
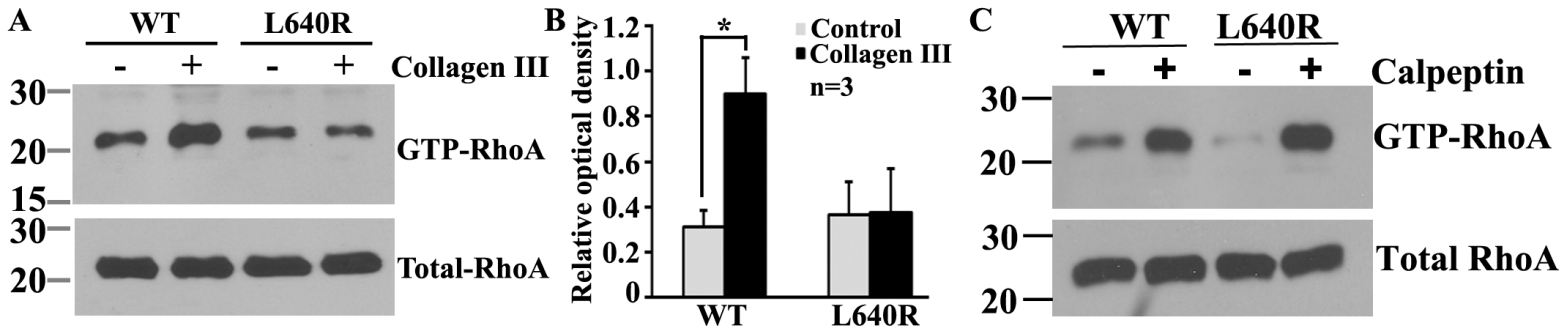
L640R mutation attenuates RhoA activation upon collagen III stimulation. (**A**) The addition of collagen III caused an increased level of GTP-RhoA in cells transfected with wild type GPR56, but not in cells transfected with L640R mutant. Total RhoA expression in the cell lysate served as a loading control. (**B**) Bar graph of the relative optical density of GTP-RhoA. n = 3, *P = 0.024. (**C**) The addition of Calpeptin resulted in a comparable elevation of GTP-RhoA in cells transfected either wild type or L640R mutant *Gpr56* cDNA.

Thus far, L640R appears to behave like the wild type receptor in our assays, despite the fact that it confers a deleterious null phenotype in humans [7], [24]. We therefore hypothesized that the single amino acid change from Leucine to Arginine in the seventh transmembrane spanning abolishes the signaling capacity of the receptor upon ligand binding. To test this hypothesis, we performed a RhoA activation assay. HEK 293T cells were transfected with either wild type or L640R mutant *Gpr56* cDNA. The transfected cells were treated with either acetic acid or collagen III for 5 minutes, followed by GTP-Rho pull-down assay as previously described [10]. Indeed, cells transfected with L640R mutant cDNA failed to induce RhoA activation upon ligand stimulation, in contrast to the robust RhoA activation in cells transfected with wild type GPR56 (Fig. 4A and B). To further confirm that the lack of RhoA activation associated with L640R mutant is GPR56 signaling specific, we measured RhoA activation in cells expressing wild type or L640R upon the addition of RhoA activator Calpeptin. L640R transfected cells demonstrated a robust RhoA activation upon Calpeptin treatment comparable to cells transfected with wild type GPR56 (Fig. 4C). In sum, the BFPP-associated L640R mutation in GPR56^C^ abolishes GPR56 function by disrupting downstream RhoA signaling.

### L640, an Evolutionarily Conserved Amino Acid Residue, is Important for GPR56 Signaling

**Figure 5:**
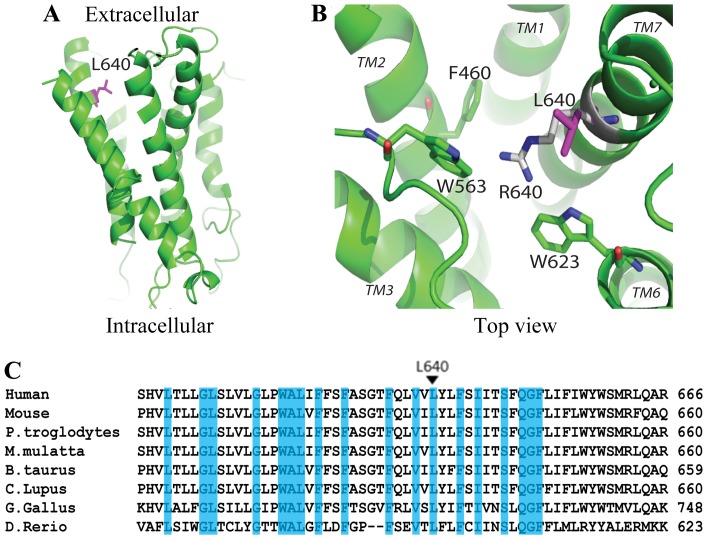
Position of L640 in the GPR56 transmembrane helices. (**A**) L640 is positioned on the transmembrane helix 7 close to the extracellular side. Its side chain faces the extracellular cavity where ligands typically bind to in other receptor families. L640 is colored magenta. (**B**) The mutation of Leucine to Arginine creates a long charged side chain that may reside in multiple conformations. The side chain of Arginine (white and blue) is able to reach residues from other transmembrane helices and possibly be involved in new interactions that the Leucine side chain is unable to. These interactions may favor a locked inactive conformation of the receptor. The GPR56 model was made by MODELLER based on the secretin family structure (PDB ID: 4L6R). Nitrogen and oxygen atoms are colored blue and red, respectively. Figure was drawn by PYMOL. (**C)** Alignment of the TM7 in GPR56 orthologs. ClustalW was used to perform an amino acid sequence alignment of the TM7 in GPR56 orthologs. L640 is highly conserved among GPR56 orthologs.

**Figure 6:**
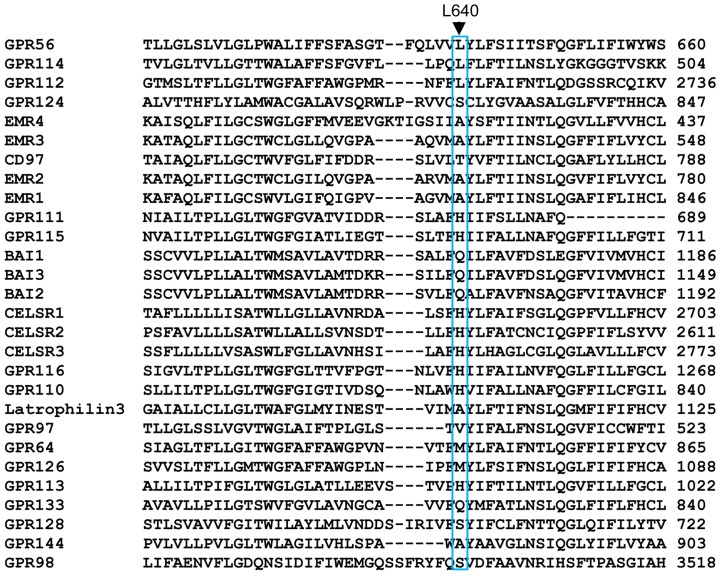
Alignment of TM7 in adhesion-GPCRs. ClustalW was used to perform an amino acid sequence alignment of the L640 residue in adhesion-GPCRs. L640 is not conserved among the majority of adhesion GPCRs.

Crystal structures of multiple GPCRs from the rhodopsin and secretin families have been determined [25]–[28]. These structures shed light on how GPCRs are activated, and indicate that there are major differences among the different GPCR families, especially in the ligand binding extracellular cavity. While structural information for the transmembrane helices of adhesion GPCRs has yet to be determined, computational modeling of GPR56 showed that L640 is positioned in the last transmembrane helix close to the extracellular side (Fig. 5A). The L640 side chain faces the extracellular cavity (Fig. 5B), which is important for ligand interaction in the rhodopsin and secretin families of GPCRs. Amino acid sequence alignment revealed that L640 is evolutionarily conserved in GPR56, across multiple species, but not so in the majority of other adhesion GPCRs family members (Fig. 5C and Fig. 6).

## Discussion

**Figure 7:**
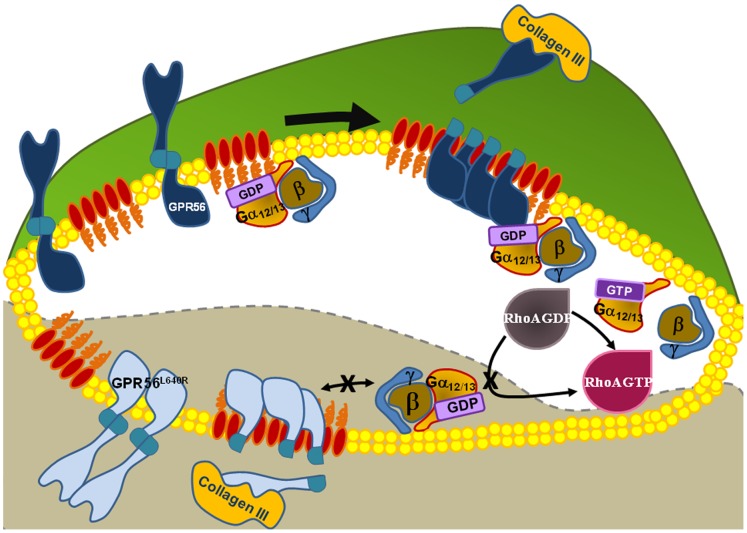
The signaling of GPR56. The binding of collagen III with wild type GPR56 (dark blue) releases GPR56^N^ from the membrane-bound GPR56^C^ and triggers the association of GPR56^C^ with lipid rafts, thus activating its downstream signaling molecular RhoA. For the L640R mutant (light blue), the binding of collagen III to the receptor fails to couple to Gα_12/13_ and activate RhoA, despite its ability to release GPR56^N^ from the membrane-bound GPR56^C^ and to trigger the association of GPR56^C^ with lipid rafts.

We previously showed that Collagen III is a ligand of GPR56 in the developing brain [10], [29]. Upon binding to collagen III, GPR56 activates RhoA via coupling to Gα_12/13_ [10]. Here, we discover that collagen III binding also induces release of the GPR56^N^ fragment, allowing the GPR56^C^ fragment to associate with DRMs (and presumably lipid rafts *in vivo*). Surprisingly, the L640R mutation does not inhibit these processes, but instead blocks downstream RhoA activation (Fig. 7).

Like most other adhesion GPCRs, GPR56 is autocatalytically cleaved through the GPS motif between amino acids histidine-381 and leucine-382 into N- and C-terminal fragments, GPR56^N^ and GPR56^C^, respectively [8], [12], [30]. Although mutations in the GPS domain disrupt this cleavage and cause human BFPP disease, the biological significance of this cleavage is not entirely clear. We previously showed that the cleaved GPR56^N^ remains associated with GPR56^C^ at the plasma membrane [12]. Furthermore, work from Hall’s group showed that overexpression of GPR56^C^ alone results in constitutive activation of RhoA [23]. We therefore hypothesized that the association of GPR56^N^ and GPR56^C^ keeps the receptor in an inactivated state, and the binding of collagen III activates the receptor by removing GPR56^N^ from GPR56^C^. Indeed, our data show that collagen III treatment cause the releases of GPR56^N^ from cell surface and the activation of RhoA.

Lipid rafts are special microdomains on the cell plasma membrane, composed of a combination of sphingolipids, cholesterol, and membrane proteins. These specialized membranes mediate cellular processes by serving as organizing centers for the assembly of signaling molecules, influencing membrane protein trafficking, and regulating neurotransmission. Many membrane-localized signaling pathways have been reported to depend on association with lipid rafts including those activated by EGF, IgE, the T- and B-cell receptors, and CD40-mediated Akt phosphorylation [31]. A recent report showed a dynamic residence of the myeloid cell-specific adhesion GPCR EMR2 during signaling [32]. In this study, we demonstrated that collagen III treatment causes a shift of GPR56^C^ from non-raft to raft fractions, suggesting that GPR56 probably signals most efficiently in these nanodomains. In contrary to the previous report, we also observed a similar shift in L640R mutant receptors upon ligand stimulation (Fig. 3).

*In vitro* characterization of GPR56 indicates that various BFPP-associated mutations disrupt its function through different mechanisms. Mutations in the tip of GPR56^N^ renders the receptor inactive by abolishing ligand binding [16], [22], whereas mutations at the GPS motif within the GAIN domain disrupt receptor function by abolishing the GAIN domain-mediated receptor autocleavage [12]. Previous biochemical studies have demonstrated that most disease-associated mutations reduce the surface expression of GPR56, with the exception of the L640R mutant that actually retains a high level of surface expression [12]. This reinforces the reasoning that there are probably other mechanisms responsible for the null phenotype associated with this mutation. In this study, we discovered that L640R mutant receptor behaves very similarly to the wild type GPR56 except in regards to collagen III-mediated RhoA activation. One possible explanation is that the L640R mutation disrupts coupling with Gα_12/13_, thereby abolishing RhoA signaling capabilities. As we did not directly measure collagen III binding to the L640R mutant, it is also formally possible that collagen III binding is compromised by this mutation, thereby blocking RhoA signal transduction. This seems implausible as collagen III treatment released GPR56^N^ from the membrane-bound GPR56^C^ as well as triggered a shift of GPR56^C^ from non-DRM to DRM fractions in both wild type and L640R mutant GPR56.

The L640 side chain faces the extracellular cavity (Fig. 5B), which is important for ligand interaction in the rhodopsin and secretin families of GPCRs. Amino acid sequence alignment revealed that L640 is evolutionarily conserved in GPR56, across multiple species, but not so in the majority of other adhesion GPCRs family members (Fig. 5C and Fig. 6). Taken together, it is possible that the mutation of Leucine to an Arginine may interfere with the activation of the receptor by creating a locked inactive receptor. The long and charged side chain of arginine may reach out to residues from other transmembrane helices of the receptor and become involved in new interactions that favor an inactive receptor conformation, abolishing the signaling ability of the receptor (Fig. 5B). Alternatively, L640 could be critical for Gα_12/13_ docking to GPR56C, thereby rendering L640R incapable of signaling via RhoA.
